# Clinical significance of neutrophil to lymphocyte ratio in ischemic stroke and transient ischemic attack in young adults

**DOI:** 10.1186/s12883-022-03011-7

**Published:** 2022-12-14

**Authors:** Yanfang Liu, Guangshuo Li, Jiaokun Jia, Xinmin Liu, Jiahuan Guo, Xingquan Zhao

**Affiliations:** 1 Department of Neurology, Beijing Tiantan Hospital, Capital Medical University, Beijing, China; 2 China National Clinical Research Center for Neurological Diseases, Beijing, China; 3 Research Unit of Artificial Intelligence in Cerebrovascular Disease, Chinese Academy of Medical Sciences, Beijing, China; 4 Beijing Institute of Brain Disorders, Collaborative Innovation Center for Brain Disorders, Capital Medical University, Beijing, China

**Keywords:** Stroke, Neutrophil, Inflammation

## Abstract

**Objective:**

Few studies evaluated the association between neutrophil to lymphocyte ratio (NLR) and clinical outcomes in ischemic stroke or transient ischemia attack (TIA) in young adults. We aimed to investigate the relationship of NLR with 90-day functional independence in ischemic stroke or TIA in young adults.

**Methods:**

We retrospectively included patients aged 18–45 and diagnosed with ischemic stroke or TIA. Information including demographics, clinical and imaging characteristics, and the 90-day clinical outcome was collected. The primary outcome was excellent clinical outcome at 90 days, defined as mRS 0–1. Logistic regression analyses and a receiver operator characteristic (ROC) curve were used to investigate the association between NLR and 90-day clinical outcome.

**Results:**

A total of 691 young patients with ischemic stroke or TIA were included in the final study. A higher level of NLR indicated poorer clinical outcome at 90 days (p for trend <0.001). The multivariable logistics regression suggested that NLR was an independent predictor of mRS 0–1 at 90 days (crude OR: 0.88, 95% CI 0.83–0.94, *p* < 0.001; adjusted OR of model 2: 0.87, 95% CI 0.84–0.94, p < 0.001; adjusted OR of model 3: 0.92, 95% CI 0.84–0.99, *p* = 0.04).

**Conclusion:**

In our study, a higher level of NLR was correlated with poorer functional outcomes at 90 days in ischemic stroke or TIA in young adults.

## Introduction

Ischemic stroke in young adults caused a great global burden of disease in the world with incidences from 5 to 40 per 100,000 person-years across different regions in the world [1]. Given the differences in clinical features, etiology, and risk factors of juvenile ischemic stroke compared to ischemic stroke in the elderly population [2], young ischemic stroke deserves more investigations to further reveal the mechanism and interventions.

The inflammatory process initiated after the onset of ischemic stroke was related to the progression and prognosis of ischemic stroke [3–5]. Plus, preceding and post-stroke infections in young adults might also be associated with clinical outcomes in young ischemic stroke [6]. Some laboratory parameters including leukocyte rate, which were associated with inflammation and infection, were found to be related to clinical outcomes in young ischemic stroke [7]. Neutrophil to lymphocyte ratio (NLR), an inflammatory marker, was also reported to be correlated with clinical outcomes in patients irrespective of age with ischemic stroke [8, 9]. NLR is also a quite convenient biomarker in the clinical practice for neutrophil and lymphocyte count could be obtained through a routine laboratory examination. However, it remained unknown whether the role of NLR in young ischemic stroke was the same as that in the elderly patients with ischemic stroke. Hence, it is necessary to investigate the clinical significance of NLR and other inflammatory parameters in young ischemic stroke, considering different rate or role of pre- or post- stroke infection.

The aim of our study was to investigate the relationship between NLR and 90-day functional outcomes in ischemic stroke and TIA in young adults.

## Methods

### Study design

Patients admitted to the neurology department of Beijing Tiantan Hospital were screened for eligibility in our retrospective study. The study was approved by the ethics committee of Beijing Tiantan Hospital.

### Participants

Young patients with ischemic stroke or TIA were recruited consecutively from January 2019 to December 2021 and would be assessed for eligibility if they met the inclusion criteria:

1) aged 18–45 years;

2) ischemic stroke or TIA;

3) admitted within 3 days from ischemia onset.

Patients were excluded if they were 1) diagnosed with cerebral venous thrombosis, iatrogenic stroke; 2) without laboratory test results on neutrophils or lymphocytes; 3) disability before the index event of stroke or TIA (mRS ≥2); 4) without information on 90-d mRS.

### Data collection and outcome

A neurological resident physician (L.G.) collected clinical data blinded to the results of blood tests and imaging information. The clinical data collected included demographic information (age, sex), medical history (hypertension, diabetes, hyperlipidemia, prior stroke, etc.), and alcohol intake / smoking status. Neurological impairment severity was evaluated by the National Institute of Health Stroke Scale (NIHSS) score [10]. Stroke etiology was classified based on the Trial of Org 10,172 in Acute Stroke Treatment (TOAST) classification [11].

Another neurologist (J.J.) conducted the data collection of imaging and laboratory examination. NLR was calculated by neutrophil count divided by lymphocyte count. Peripheral blood samples were collected within 24 hours from admission. Excellent functional outcome was defined as modified Rankin Scale (mRS) 0–1 at 90 days.

### Statistical analysis

The included patients were categorized into four groups according to the NLR level (Q1-Q4 based on median and interquartile ranges). For continuous variables, normally distributed data were shown as mean ± SD and compared using the student *t* test while non-normally distributed data were shown as median (interquartile range) and compared using Mann–Whitney U test. The normality was tested by the Shapiro test. Classified variables were shown as number (percentage) and compared using the χ2 or Fisher’s exact tests. Adjusted confounders in multivariable logistics analyses were variables with a *P* value < 0.05 in baseline comparisons. All statistical analyses were performed with the SPSS 25.0. *P* value< 0.05 was defined as statistically significant.

## Results

From January 2019 to December 2021, 691 young patients were included in our study. The included patients had an average age was 37 [33–42] and 83.94% (580/691) of them were male. Among the included patients, 4.49% (31/691) patients were treated with intravenous thrombolysis (IV tPA). The median admission NIHSS score was 2 (0–5) and 14.62% (101/691) patients had hemorrhagic transformation during hospitalization. The proportion of 77.71% (537/691) patients had excellent functional outcome (mRS 0–1) at 90 days.

### Baseline comparison between the Q1-Q4 groups of NLR

**Fig. 1:**
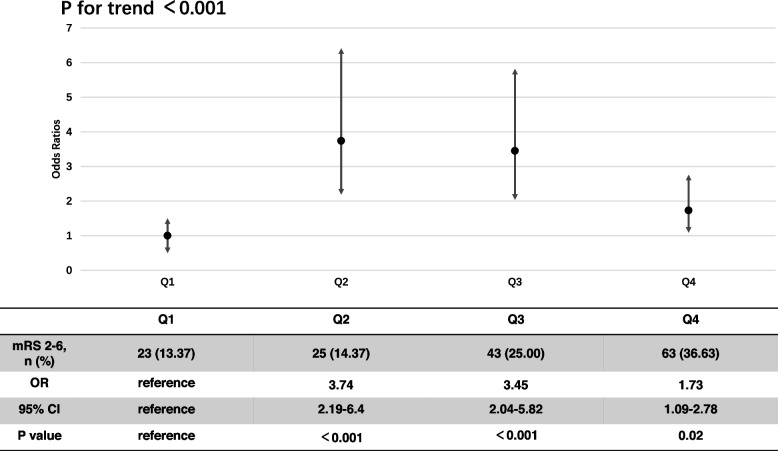
Relationship between different level of NLR and 90d disability (mRS 2–6)

Compared with the Q1-Q3 groups (Table 1), the Q4 group had a larger proportion of IV tPA (8.72%, *p* = 0.008), higher level of systolic blood pressure (148 [132.75–170] mmHg, *p* < 0.001) as well as a higher level of diastolic blood pressure (94.5 [82–110] mmHg, *p* < 0.001), more severe neurological impairment (median NIHSS score, 3.5 [1–7], *p* < 0.001) and a higher proportion of pulmonary infection (11.05%, p < 0.001). The Q4 group also had a higher proportion of disability at 90 days (mRS 2–6, 36.63%, p < 0.001). As Fig. 1 showed, proportions of disability increased among the Q1-Q4 groups (P for trend < 0.001).

**Table 1:** baseline comparison between NLR Q1-Q4 groups

	Overall *N* = 691	Q1 *N* = 172	Q2 *N* = 174	Q3 N = 172	Q4 N = 172	P value
Age, years (median [IQR])	37 [33, 42]	37 [32, 41]	37 [33, 41]	38 [33, 42]	38 [34, 42]	0.07
Male, n (%)	580 (83.94)	148 (86.05)	153 (87.93)	140 (81.40)	138 (80.23)	0.16
Admission NIHSS score, (median [IQR])	2 [0, 5]	1 [0, 3]	1 [0, 4]	2 [1, 5]	3.5 [1, 7]	< 0.001
Thrombolysis, n (%)	31 (4.49)	2 (1.16)	7 (4.02)	7 (4.07)	15 (8.72)	0.01
Hypertension, n (%)	324 (46.89)	66 (38.37)	82 (47.13)	90 (52.33)	85 (49.42)	0.06
Diabetes, n (%)	120 (17.37)	28 (16.28)	32 (18.39)	35 (20.35)	24 (13.95)	0.45
Dyslipidemia, n (%)	91 (13.17)	25 (14.53)	23 (13.22)	19 (11.05)	24 (13.95)	0.79
Atrial fibrillation, n (%)	11 (1.59)	3 (1.74)	1 (0.57)	4 (2.33)	3 (1.74)	0.62
Prior ischemic stroke, n (%)	74 (10.71)	15 (8.72)	16 (9.20)	28 (16.28)	15 (8.72)	0.06
Prior hemorrhagic stroke, n (%)	9 (1.30)	3 (1.75)	2 (1.15)	1 (0.58)	2 (1.16)	0.79
Coronary heart disease, n (%)	22 (3.18)	4 (2.33)	6 (3.45)	6 (3.49)	6 (3.49)	0.91
Smoking status, n (%)						0.75
Never smoker	302 (43.70)	69 (40.12)	75 (43.10)	80 (46.51)	77 (44.77)	
Past smoker	22 (3.18)	8 (4.65)	4 (2.30)	6 (3.49)	4 (2.33)	
Current smoker	367 (53.11)	95 (55.23)	95 (54.60)	86 (50.00)	91 (52.91)	
Alcoholic status, n (%)						0.09
Never drinked	475 (68.74)	103 (59.88)	128 (73.56)	123 (71.51)	120 (69.77)	
Past drinked	11 (1.59)	5 (2.91)	2 (1.15)	3 (1.74)	1 (0.58)	
Current drinking	205 (29.67)	64 (37.21)	44 (25.29)	46 (26.74)	51 (29.65)	
Prior antiplatelet agents, n (%)	52 (7.53)	8 (4.65)	15 (8.62)	15 (8.72)	14 (8.14)	0.43
Prior anticoagulation agents, n (%)	11 (1.59)	2 (1.16)	1 (0.57)	4 (2.33)	4 (2.33)	0.47
Systolic blood pressure, mmHg (median [IQR])	141.00 [129.00, 161.00]	134.50 [124.00, 149.00]	145.00 [131.25, 160.00]	143.00 [132.00, 164.00]	148.00 [132.75, 170.00]	< 0.001
Diastolic blood pressure, mmHg (median [IQR])	91.00 [81.00, 104.00]	86.50 [78.00, 96.25]	91.50 [80.25, 104.00]	94.00 [84.75, 105.00]	94.50 [82.00, 110.00]	< 0.001
Triglyceride, mmol/L (median [IQR])	1.42 [1.03, 2.04]	1.46 [1.14, 2.15]	1.40 [1.03, 2.05]	1.52 [1.06, 2.20]	1.33 [0.99, 1.79]	0.1
Cholesterol, mmol/L (median [IQR])	3.74 [3.03, 4.53]	3.74 [3.12, 4.48]	3.60 [3.02, 4.43]	3.87 [3.08, 4.56]	3.83 [3.12, 4.64]	0.55
High density lipoprotein, mmol/L (median [IQR])	0.99 [0.85, 1.16]	1.03 [0.86, 1.17]	0.95 [0.82, 1.12]	0.98 [0.85, 1.13]	1.03 [0.86, 1.19]	0.06
Low density lipoprotein, mmol/L (median [IQR])	2.18 [1.63, 2.90]	2.18 [1.63, 2.87]	2.13 [1.62, 2.79]	2.25 [1.54, 3.01]	2.22 [1.71, 3.10]	0.62
CRP, mg/L (median [IQR])	1.18 [0.49, 3.66]	0.84 [0.33, 2.07]	1.01 [0.44, 2.77]	1.12 [0.52, 3.48]	2.57 [0.82, 9.48]	< 0.001
NLR (median [IQR])	2.60 [1.91, 3.85]	1.54 [1.25, 1.71]	2.21 [2.07, 2.39]	3.18 [2.89, 3.46]	5.51 [4.40, 7.82]	< 0.001
Glucose, mmol/L (median [IQR])	5.50 [4.70, 6.90]	5.00 [4.48, 6.10]	5.50 [4.57, 6.80]	5.65 [4.85, 7.04]	6.08 [5.20, 7.39]	< 0.001
Pulmonary infection, n (%)	32 (4.63)	1 (0.58)	3 (1.72)	9 (5.23)	19 (11.05)	< 0.001
Urinary infection, n(%)	5 (0.72)	0 (0.00)	0 (0.00)	3 (1.74)	2 (1.16)	0.14
Infectious diarrhea, n (%)	5 (0.72)	0 (0.00)	1 (0.57)	2 (1.16)	2 (1.16)	0.53
Deep venous thrombosis, n (%)	20 (2.89)	4 (2.33)	5 (2.87)	3 (1.74)	8 (4.65)	0.41
Hemorrhagic transformation, n (%)	101 (14.62)	20 (11.63)	22 (12.64)	26 (15.12)	33 (19.19)	0.20
TOAST, n (%)						0.09
LAA	314 (45.44)	76 (44.19)	76 (43.68)	86 (50.00)	76 (44.19)	
CE	46 (6.66)	10 (5.81)	5 (2.87)	16 (9.30)	15 (8.72)	
SAA	80 (11.58)	21 (12.21)	24 (13.79)	15 (8.72)	19 (11.05)	
Other	150 (21.71)	37 (21.51)	36 (20.69)	31 (18.02)	46 (26.74)	
Unknown	101 (14.62)	28 (16.28)	33 (18.97)	24 (13.95)	16 (9.30)	
mRS score at 90 days (median [IQR])	0.00 [0.00, 1.00]	0.00 [0.00, 1.00]	0.00 [0.00, 1.00]	1.00 [0.00, 1.25]	1.00 [0.00, 2.00]	< 0.001
mRS 0–1, n (%)	537 (77.71)	149 (86.63)	149 (85.63)	129 (75.00)	109 (63.37)	< 0.001

NLR, neutrophil to lymphocyte ratio; NIHSS, national institutes of health stroke scale; TOAST, Trial of Org 10,172 in Acute Stroke Treatment; LAA, large atherosclerosis artery; CE, cardiac embolism; SAA, small artery occlusion; mRS, modified Rankin Scale

### Relationship between NLR and functional outcome at 90 days

**Fig. 2:**
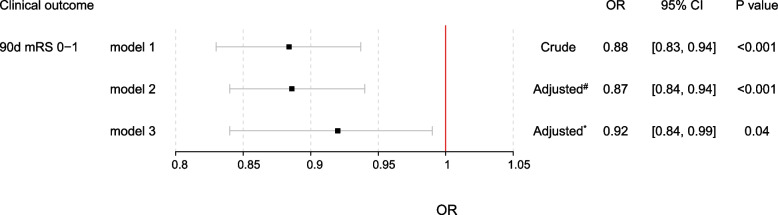
Multivariable logistics regression analyses: #) adjusted for age and sex; *) adjusted for age, random glucose, hemorrhagic transformation, systolic blood pressure, diastolic blood pressure, smoke status, alcoholic status, history of dyslipidemia, history of atrial fibrillation, pulmonary infection, low-density lipoprotein, CRP and NIHSS score

Table 2 summarized the comparison of baseline characteristics between mRS 2–6 and mRS 0–1 group. Compared to mRS 2–6 group, mRS 0–1 group was younger (37 [32–42] vs. 38 [35–42], *p* = 0.03), had a lower proportion of hemorrhagic transformation (12.48% vs. 22.08%, *P* = 0.004), higher level of random glucose (5.46 [4.62–6.71] vs. 5.92 [5.00–7.68], *p* = 0.002), lower level of systolic blood pressure (140 [129–159] vs. 149 [129.25–170], *p* = 0.006) as well as lower level of diastolic blood pressure (90 [81–103] vs. 96 [82, 109.50], *p* = 0.008), higher proportion of dyslipidemia (85.29% vs. 92.21%, *p* = 0.035), lower proportion of atrial fibrillation (0.93% vs. 3.9%, *p* = 0.026), lower proportion of current smoker (50.28% vs. 62.99%, *p* = 0.017), lower admission NIHSS score (1 [0–3] vs. 8 [6–10], *p* < 0.001), lower proportion of pulmonary infection (2.79% vs. 11.04%, *p* < 0.001), lower level of low-density lipoprotein (2.13 [1.60–2.87] vs. 2.36 [1.81–3.00], *p* = 0.035), lower level of CRP (1.01 [0.43–2.93] vs. 2.17 [0.90–7.14], p < 0.001) and lower level of NLR (2.39 [1.84–3.54] vs. 3.38 [2.48–5.24], *p* < 0.001). As shown in Fig. 2*,* multivariable logistics regression analyses found that NLR indicated to be an independent predictor of mRS 0–1 at 90 days (crude OR: 0.88, 95% CI 0.83–0.94, *p* < 0.001; adjusted OR of model 2: 0.87, 95% CI 0.84–0.94, p < 0.001; adjusted OR of model 3: 0.92, 95% CI 0.84–0.99, *p* = 0.04).

**Table 2:** baseline comparison between mRS 2–6 and mRS 0–1 groups

	Overall N = 691	mRS 2–6 *N* = 154	mRS 0–1 *N* = 537	P value
Age, years (median [IQR])	37 [33, 42]	38 [35, 42]	37 [32, 42]	0.03
Male, n (%)	111 (16.06)	20 (12.99)	91 (16.95)	0.24
Admission NIHSS score, (median [IQR])	2 [0, 5]	8 [6, 10]	1 [0, 3]	< 0.001
Thrombolysis, n (%)	31 (4.49)	10 (6.49)	21 (3.91)	0.17
Hypertension, n (%)	324 (46.89)	74 (48.05)	250 (46.55)	0.74
Diabetes, n (%)	121 (17.51)	26 (16.88)	95 (17.69)	0.81
Dyslipidemia, n (%)	91 (13.17)	12 (7.79)	79 (14.71)	0.03
Atrial fibrillation, n (%)	11 (1.59)	6 (3.90)	5 (0.93)	0.03
Prior ischemic stroke, n (%)	74 (10.71)	20 (12.99)	54 (10.06)	0.3
Prior hemorrhagic stroke, n (%)	9 (1.30)	4 (2.60)	5 (0.93)	0.23
Coronary heart disease, n (%)	22 (3.18)	5 (3.25)	17 (3.17)	0.91
Smoking status, n (%)				0.02
Never smoker	302 (43.70)	52 (33.77)	250 (46.55)	
Past smoker	22 (3.18)	5 (3.25)	17 (3.17)	
Current smoker	367 (53.11)	97 (62.99)	270 (50.28)	
Alcoholic status, n (%)				0.44
Never drinked	475 (68.74)	100 (64.94)	375 (69.83)	
Past drinked	11 (1.59)	2 (1.30)	9 (1.68)	
Current drinking	205 (29.67)	52 (33.77)	153 (28.49)	
Prior antiplatelet agents, n (%)	52 (7.53)	14 (9.09)	38 (7.08)	0.40
Prior anticoagulation agents, n (%)	11 (1.59)	5 (3.25)	6 (1.12)	0.14
Systolic blood pressure, mmHg (median [IQR])	141.00 [129.00, 161.00]	149.00 [129.25, 170.00]	140.00 [129.00, 159.00]	0.01
Diastolic blood pressure, mmHg (median [IQR])	91.00 [81.00, 104.00]	96.00 [82.00, 109.50]	90.00 [81.00, 103.00]	0.01
Triglyceride, mmol/L (median [IQR])	1.42 [1.03, 2.04]	1.38 [1.06, 1.91]	1.43 [1.02, 2.09]	0.68
Cholesterol, mmol/L (median [IQR])	3.74 [3.03, 4.53]	3.94 [3.29, 4.55]	3.66 [3.02, 4.52]	0.09
High density lipoprotein, mmol/L (median [IQR])	0.99 [0.85, 1.16]	0.99 [0.86, 1.13]	1.00 [0.85, 1.17]	0.65
Low density lipoprotein, mmol/L (median [IQR])	2.18 [1.63, 2.90]	2.36 [1.81, 3.00]	2.13 [1.60, 2.87]	0.04
CRP, mg/L (median [IQR])	1.18 [0.49, 3.66]	2.17 [0.90, 7.14]	1.01 [0.43, 2.93]	< 0.001
NLR (median [IQR])	2.60 [1.91, 3.85]	3.38 [2.48, 5.24]	2.39 [1.84, 3.54]	< 0.001
Glucose, mmol/L (median [IQR])	5.50 [4.70, 6.90]	5.92 [5.00, 7.68]	5.46 [4.62, 6.71]	0.01
Pulmonary infection, n (%)	32 (4.63)	17 (11.04)	15 (2.79)	< 0.001
Urinary infection, n (%)	5 (0.72)	1 (0.65)	4 (0.74)	1
Infectious diarrhea, n (%)	5 (0.72)	2 (1.30)	3 (0.56)	0.68
Deep venous thrombosis, n (%)	20 (2.89)	7 (4.55)	13 (2.42)	0.27
Hemorrhagic transformation, n (%)	101 (14.62)	34 (22.08)	67 (12.48)	0.01
TOAST, n (%)				0.1
LAA	314 (45.44)	80 (51.95)	234 (43.58)	
CE	46 (6.66)	14 (9.09)	32 (5.96)	
SAA	80 (11.58)	15 (9.74)	65 (12.10)	
Other	150 (21.71)	30 (19.48)	120 (22.35)	
Unknown	101 (14.62)	15 (9.74)	86 (16.01)	

NLR, neutrophil to lymphocyte ratio; NIHSS, national institutes of health stroke scale; TOAST, Trial of Org 10,172 in Acute Stroke Treatment; LAA, large atherosclerosis artery; CE, cardiac embolism; SAA, small artery occlusion; mRS, modified Rankin Scale

### ROC curves and ordinal analyses

**Fig. 3:**
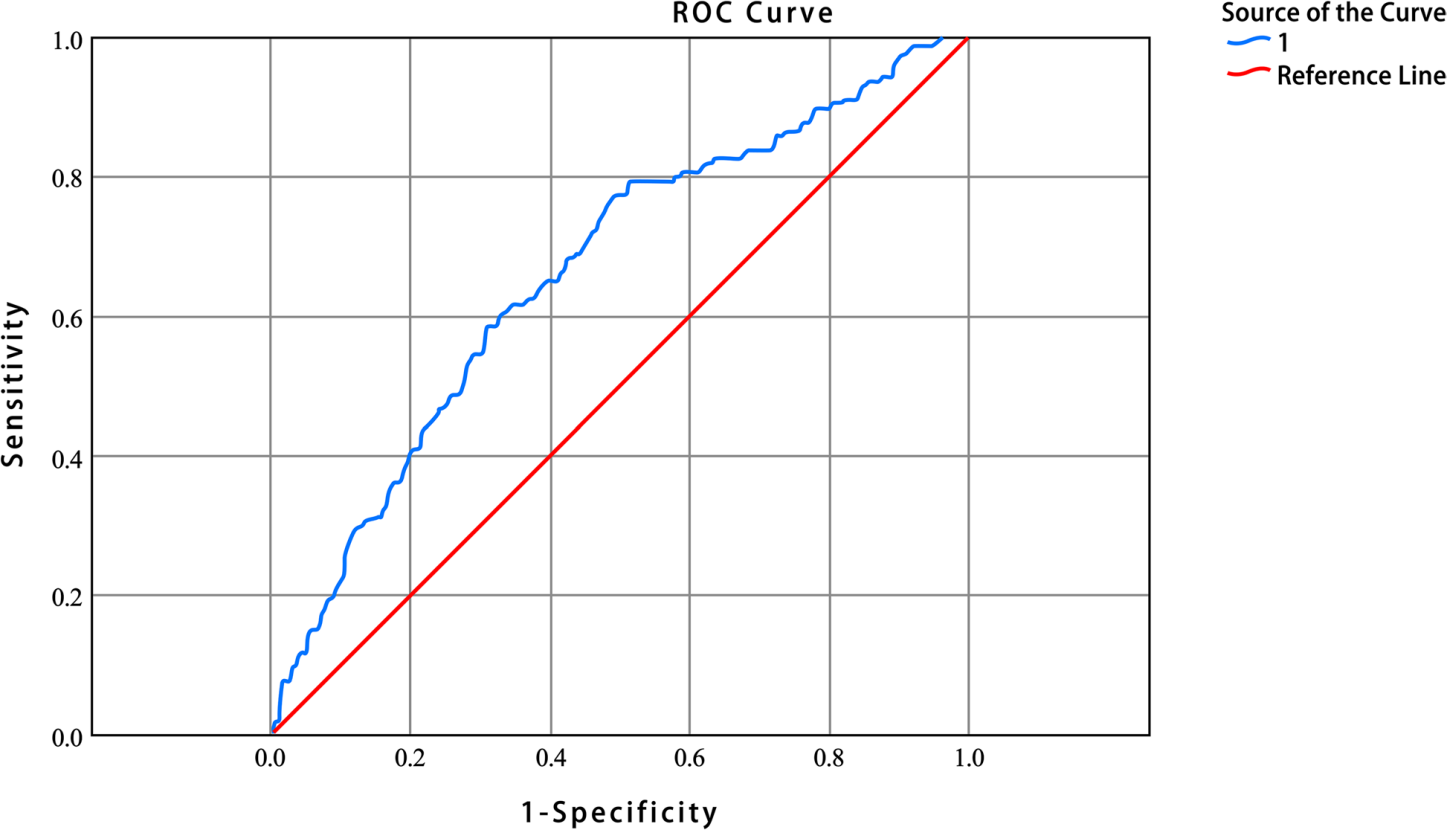
Receiver operating characteristic curves

The ROC curve **(**Fig. 3**)** showed that the area under curve (AUC) of NLR was 0.66 (95% CI 0.62–0.71, p < 0.001) and the optimal cut-off was 2.40 (sensitivity 0.77, specificity 0.50).

**Fig. 4:**
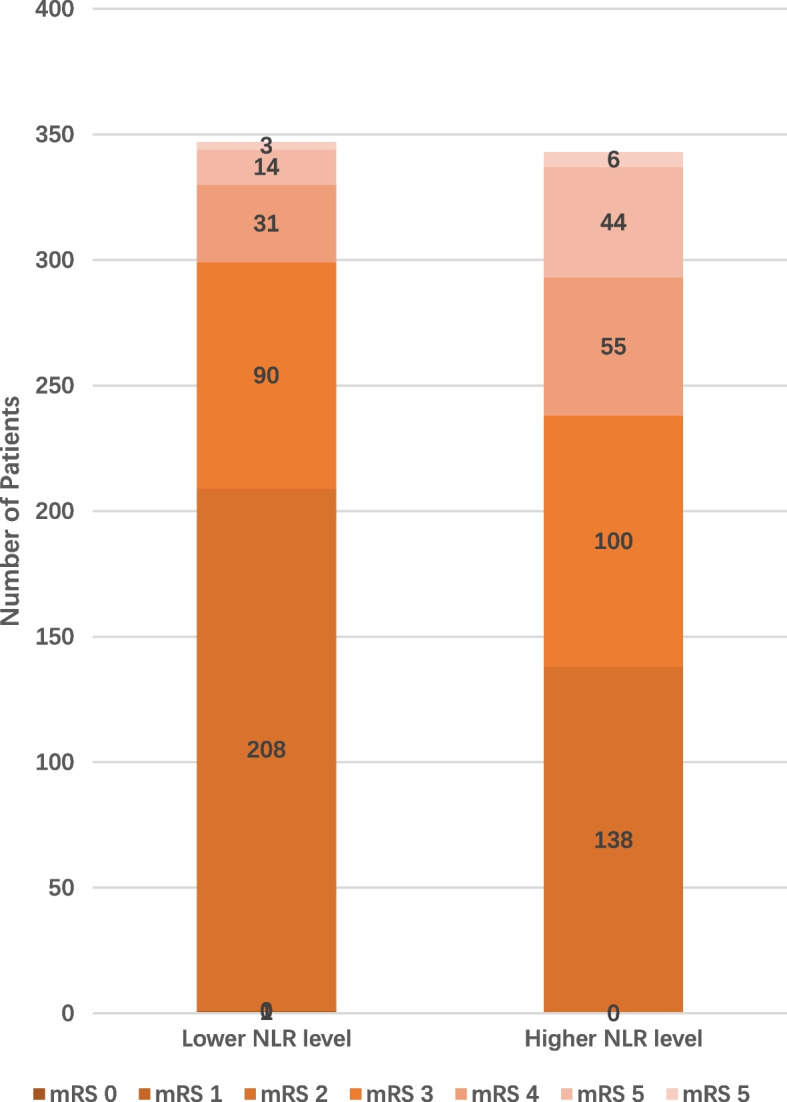
Distribution of mRS score at discharge between different level of NLR. For NLR, high level of NLR represented poor functional outcome at 90 days

**Fig. 5:**
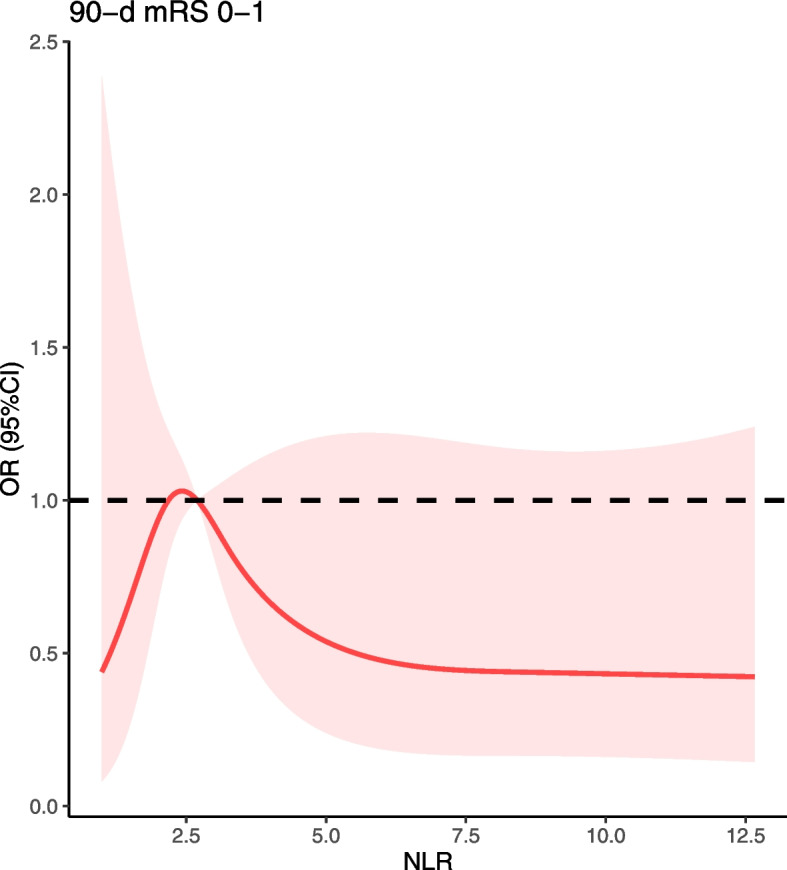
restricted cubic splines

Based on the optimal cut-off value, the shift analyses of mRS distribution between the high-level group and low-level group of NLR was shown in Fig. 4**.** For NLR, a high level of NLR represented a poor functional outcome at 90 days (*P* < 0.001). We also used restricted cubic splines (4 knots: 5th, 35th, 65th and 95th centiles) to flexibly model the association of NLR. (Fig. 5).

## Discussion

In our study, NLR was independently correlated to the functional outcome at 90 days in young patients with TIA / ischemic stroke. As far as we know, our study was the first to report the relationship between NLR and functional outcome at 90 days in young patients with TIA / ischemic stroke.

Studies based on animal models and in vitro experiments delineated the association between neuroinflammation and outcomes in ischemic stroke. Astrocytes and microglial were the initiators of neuroinflammation cascade triggered by cerebral ischemia or hypoprefusion [12, 13]. Inflammatory cytokines including interleukin-1 and tumor necrosis factor-α (TNF-α) secreted from microglia participated in recruiting immune cells including neutrophils, lymphocytes and monocytes [14, 15]. In these recruited immune cells, reactive oxygen species (ROS) build-up and activation of matrix metalloproteinases (MMP) were occurred and disrupted the integrity of the blood-brain barrier (BBB) [16]. Disrupted BBB recruited neutrophils into the brain tissue within 1 hour after ischemic stroke onset and last for about 14 days with a peak at 2–3 days [15]. The neutrophils recruited into the parenchyma generated damage to the parenchyma and worsened neurological deficits [15]. Lymphocyte were recruited into the parenchyma within days after stroke onset and both protective and harmful function was found of lymphocyte [16–18]. Lymphocytes could mend the inflammatory damage but also excrete cytotoxic chemicals at the same time [19–21]. This paradoxical function of lymphocytes may be attributed to different subtypes [22, 23]. Apart from neuroinflammation, pre- and post-stroke infections might also be associated with poor clinical outcome [6].

Our study indicated that a higher level of NLR was related to poor functional outcome at 90 days [24, 25]. However, most previous studies investigated the relationship between NLR and excellent outcome in elderly adults. Wang et al. conducted an analysis using the data of 808 patients from the Chengdu stroke registry, another registry in China, and found NLR ≥ 5 was associated with 3-month disability or death [9]. Another observational study in Germany enrolled 807 ischemic stroke patients and showed higher admission NLR level was associated with poorer functional outcome [8]. This observational study [8] also identified a similar AUC (0.69 vs. 0.66) and a higher NLR cut off value compared with our study (3.3 vs. 2.4). The higher NLR cut-off relative to that in our study was probably due to the higher admission median NLR in the Germany observational registry (3.4 vs. 2.6) [8]. Admission NLR higher than 5.9 was associated with 90-day functional outcome in patients treated with endovascular thrombectomy [26]. For patients treated with IV tPA, the cut-off value of NLR after tPA therapy was 4.8 [27].

Our study has some potential limitations. First, the retrospective study design of our observational study in a single stroke center might result in additional bias. However, blind assessment was conducted to reduce the potential bias. Secondly, the sample size of our study was limited. This study was initiated to conduct a pilot analysis to test whether the association between NLR and clinical outcome was statistical significance based on our single-center database. A future prospective registry was planned to include more participants. Third, we failed to collect the details on the ‘unknown etiology’ in TOAST of the patients enrolled in our study. Considering the retrospective design of our study, some additional examination on the rare causes of ischemic stroke was hard to perform and it was difficult to provide complete data on these rare reasons. We planned to collect more information on the rare reasons for ischemic stroke in young adults in the future prospective cohort.

## Conclusion

NLR may be served as promising biomarkers for functional outcomes at 90-day among ischemic stroke or TIA in young adults.

## Data Availability

The datasets used and/or analysed during the current study available from the corresponding author on reasonable request.
